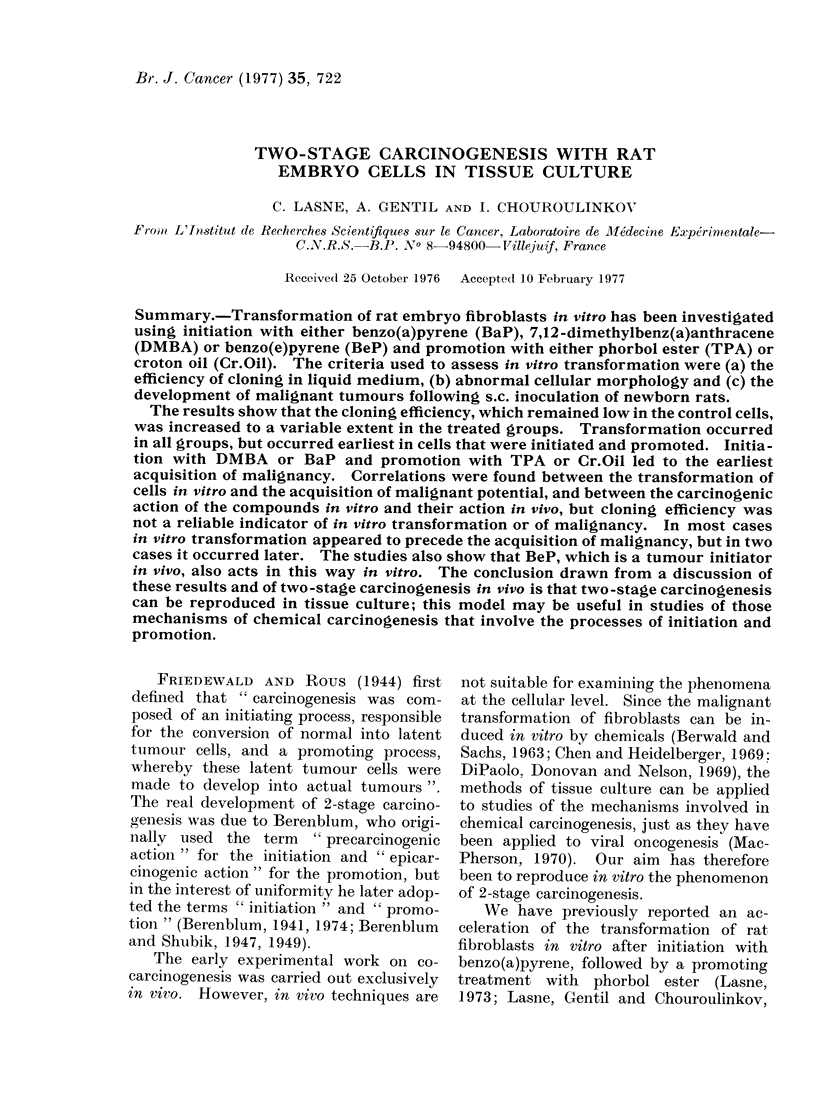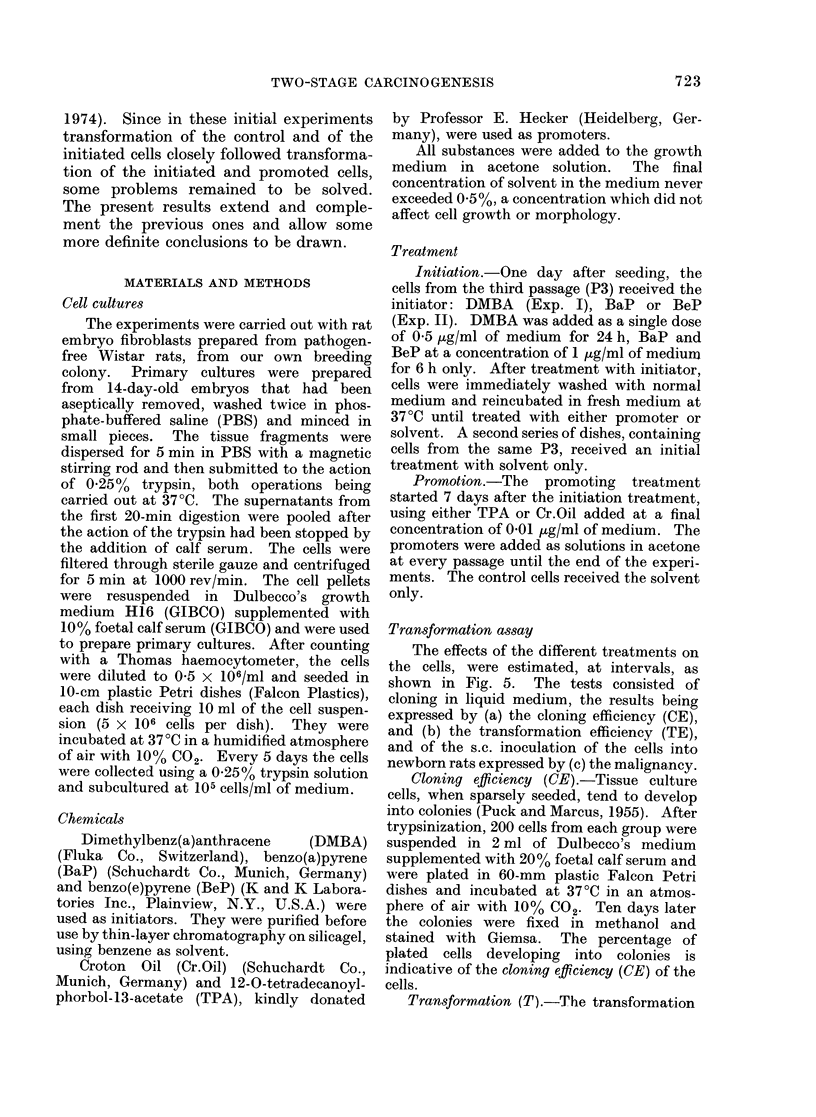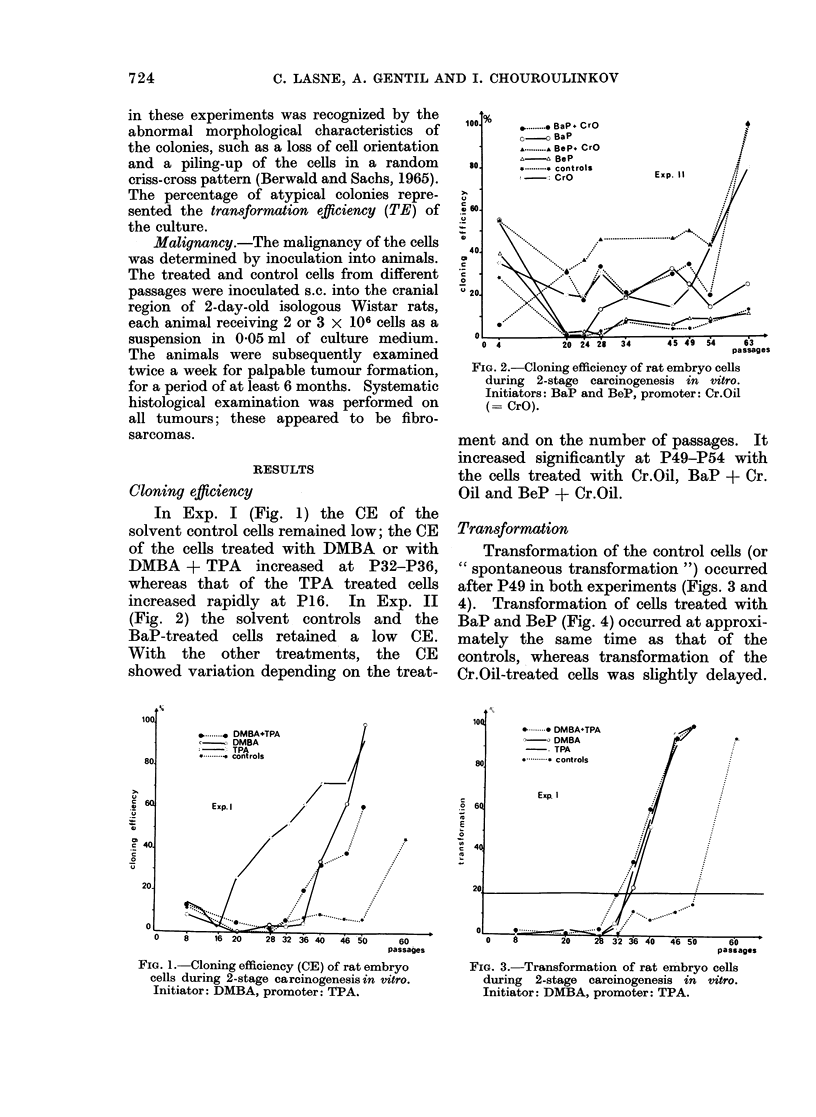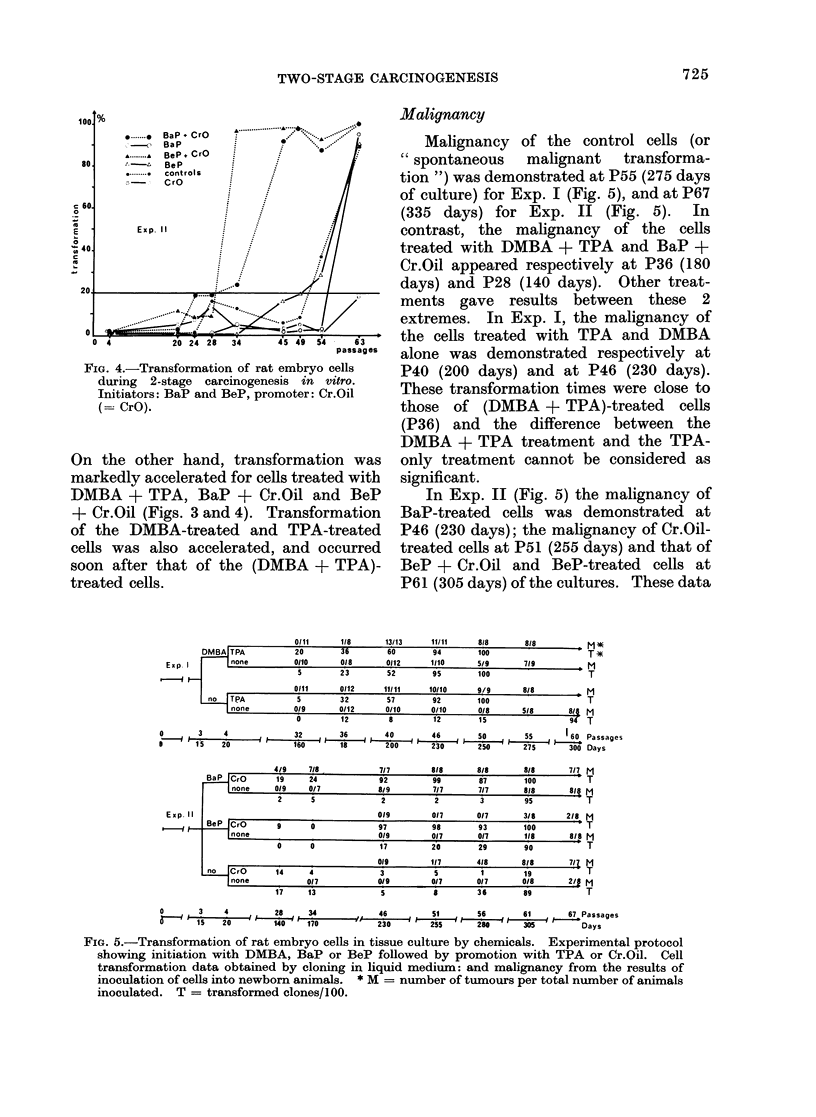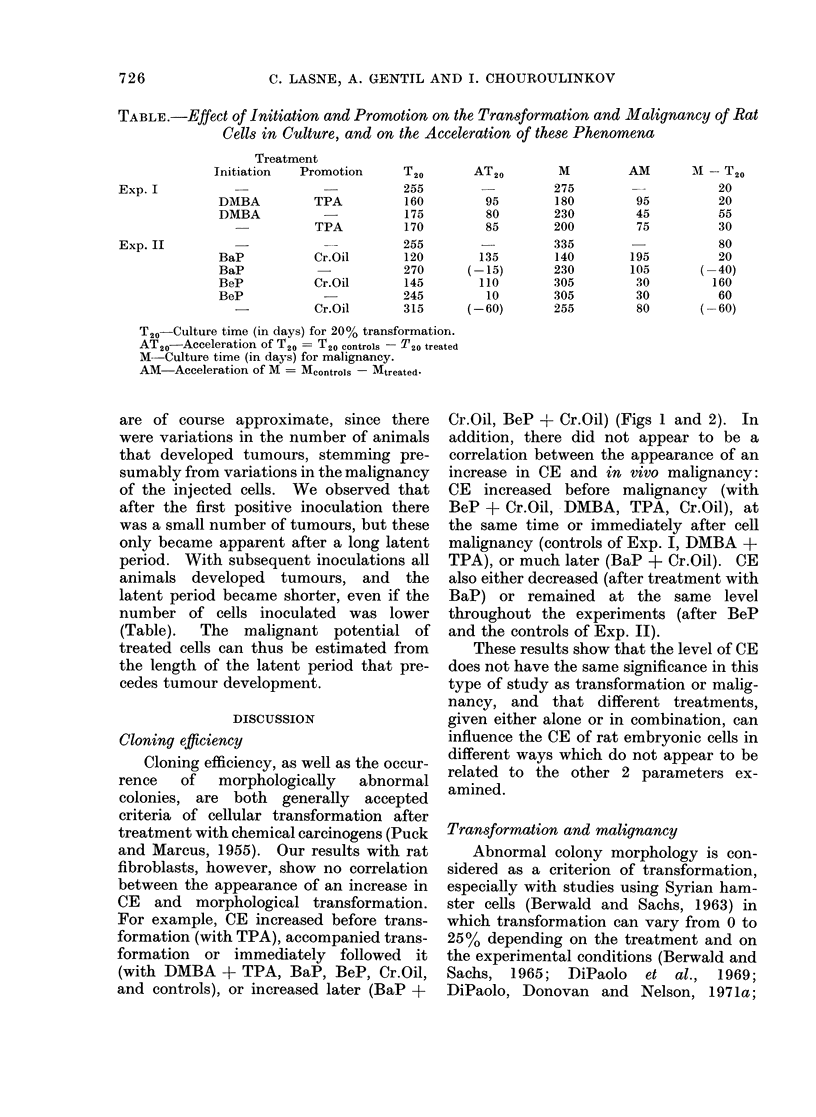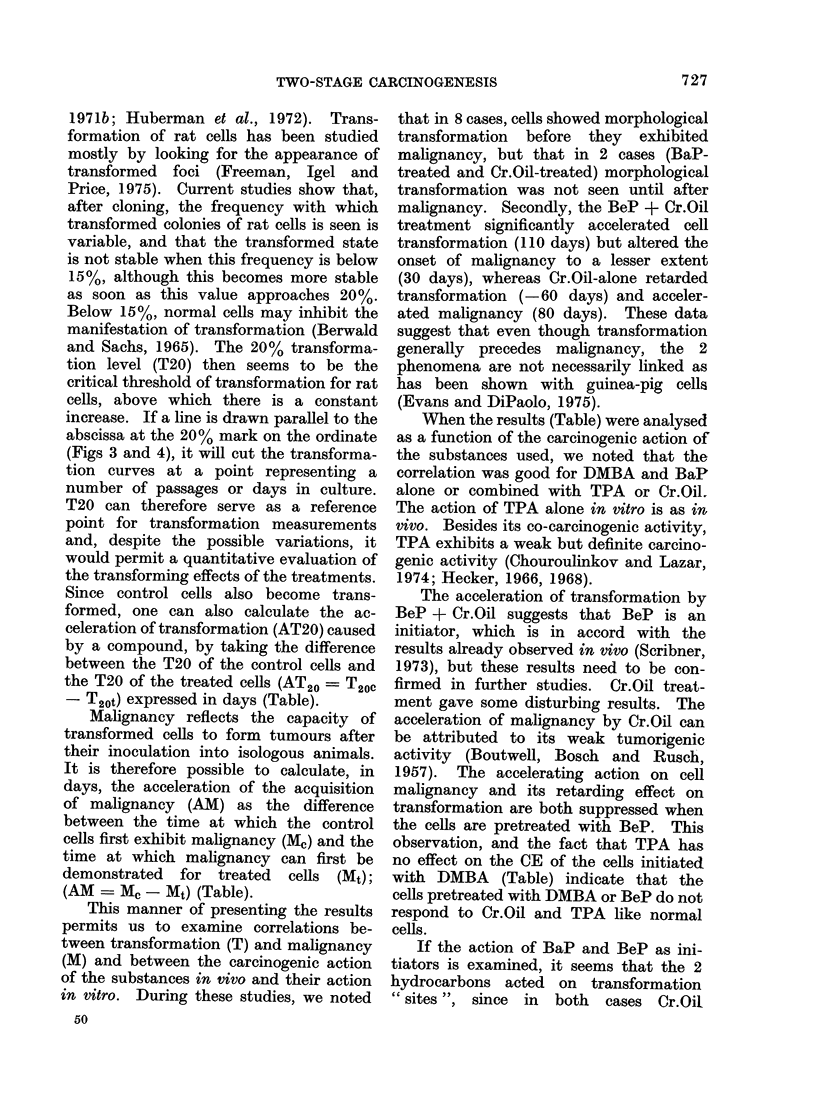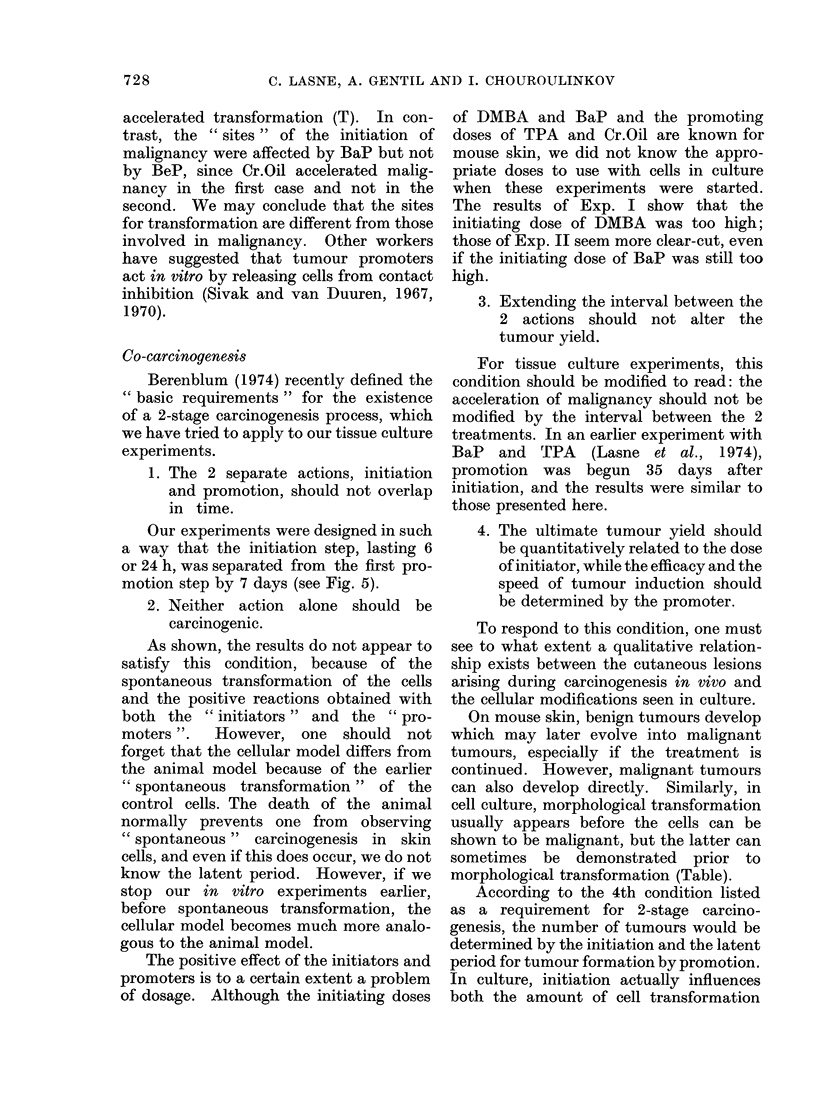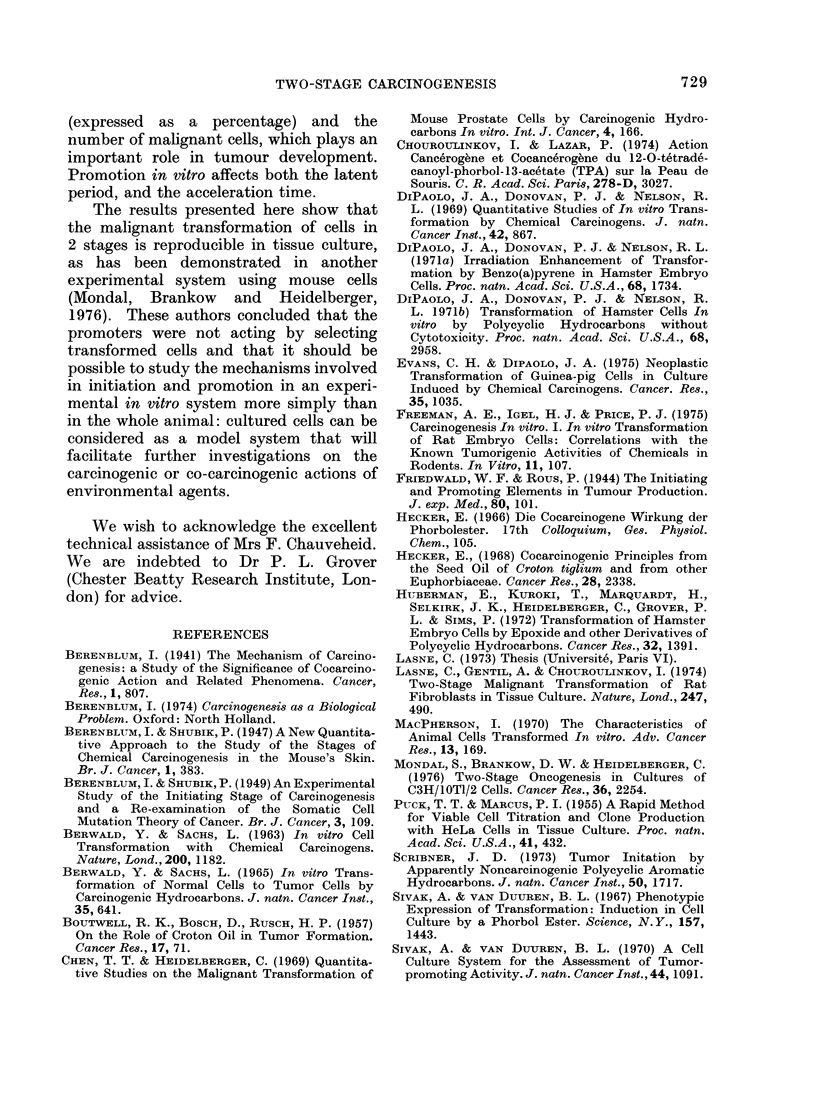# Two-stage carcinogenesis with rat embryo cells in tissue culture.

**DOI:** 10.1038/bjc.1977.113

**Published:** 1977-06

**Authors:** C. Lasne, A. Gentil, I. Chouroulinkov

## Abstract

Transformation of rat embryo fibroblasts in vitro has been investigated using initiation with either benzo(a)pyrene (BaP), 7,12-dimethylbena(a)anthracent (DMBA) or benzo(e)pyrene (BeP) and promotion with either phorbol ester (TPA) or croton oil (Cr.Oil). The criteria used to assess in vitro transformation were (a) the efficiency of cloning in liquid medium, (b) abnormal cellular morphology and (c) the development of malignant tumours following s.c. inoculation of newborn rats. The results show that the cloning efficiency, which remained low in the control cells, was increased to a variable extent in the treated groups. Transformation occurred in all groups, but occurred earliest in cells that were initiated and promoted. Initiation with DMBA or BaP and promotion with TPA or Cr.Oil led to the earliest acquisition of malignancy. Correlations were found between the transformation of cells in vitro and the acquisition of malignant potential, and between the carcinogenic action of the compounds in vitro and their action in vivo, but cloning efficiency was not a reliable indicator of in vitro transformation or of malignancy. In most cases in vitro transformation appeared to precede the acquisition of malignancy, but in two cases it occurred later. The studies also show that BeP, which is a tumour initiator in vivo, also acts in this way in vitro. The conclusion drawn from a discussion of these results and of two-stage carcinogenesis in vivo is that two-stage carcinogenesis can be reproduced in tissue culture; this model may be useful in studies of those mechanisms of chemical carcinogenesis that involve the processes of initiation and promotion.


*Br. J. Cancer* (1977) **35**, 722

### TWO-STAGE CARCINOGENESIS WITH RAT EMBRYO CELLS IN TISSUE CULTURE

C. LASNE, A. GENTIL AND I. CHOUROULINKOV

*From L'Institut de Recherches Scientifiques sur le Cancer, Laboratoire de Médecine Expérimentale— C.N.R.S.—B.P. N° 8—94800—Villejuif, France*

Received 25 October 1976 Accepted 10 February 1977

**Summary.—Transformation of rat embryo fibroblasts *in vitro* has been investigated using initiation with either benzo(a)pyrene (BaP), 7,12-dimethylbenz(a)anthracene (DMBA) or benzo(e)pyrene (BeP) and promotion with either phorbol ester (TPA) or croton oil (Cr.Oil). The criteria used to assess *in vitro* transformation were (a) the efficiency of cloning in liquid medium, (b) abnormal cellular morphology and (c) the development of malignant tumours following s.c. inoculation of newborn rats.**

**The results show that the cloning efficiency, which remained low in the control cells, was increased to a variable extent in the treated groups. Transformation occurred in all groups, but occurred earliest in cells that were initiated and promoted. Initiation with DMBA or BaP and promotion with TPA or Cr.Oil led to the earliest acquisition of malignancy. Correlations were found between the transformation of cells *in vitro* and the acquisition of malignant potential, and between the carcinogenic action of the compounds *in vitro* and their action *in vivo*, but cloning efficiency was not a reliable indicator of *in vitro* transformation or of malignancy. In most cases *in vitro* transformation appeared to precede the acquisition of malignancy, but in two cases it occurred later. The studies also show that BeP, which is a tumour initiator *in vivo*, also acts in this way *in vitro*. The conclusion drawn from a discussion of these results and of two-stage carcinogenesis *in vivo* is that two-stage carcinogenesis can be reproduced in tissue culture; this model may be useful in studies of those mechanisms of chemical carcinogenesis that involve the processes of initiation and promotion.**

FRIEDEWALD AND ROUS (1944) first defined that " carcinogenesis was composed of an initiating process, responsible for the conversion of normal into latent tumour cells, and a promoting process, whereby these latent tumour cells were made to develop into actual tumours ". The real development of 2-stage carcinogenesis was due to Berenblum, who originally used the term " precarcinogenic action " for the initiation and " epicarcinogenic action " for the promotion, but in the interest of uniformity he later adopted the terms " initiation " and " promotion " (Berenblum, 1941, 1974; Berenblum and Shubik, 1947, 1949).

The early experimental work on cocarcinogenesis was carried out exclusively *in vivo*. However, *in vivo* techniques are not suitable for examining the phenomena at the cellular level. Since the malignant transformation of fibroblasts can be induced *in vitro* by chemicals (Berwald and Sachs, 1963; Chen and Heidelberger, 1969; DiPaolo, Donovan and Nelson, 1969), the methods of tissue culture can be applied to studies of the mechanisms involved in chemical carcinogenesis, just as they have been applied to viral oncogenesis (MacPherson, 1970). Our aim has therefore been to reproduce *in vitro* the phenomenon of 2-stage carcinogenesis.

We have previously reported an acceleration of the transformation of rat fibroblasts *in vitro* after initiation with benzo(a)pyrene, followed by a promoting treatment with phorbol ester (Lasne, 1973; Lasne, Gentil and Chouroulinkov,

1974). Since in these initial experiments transformation of the control and of the initiated cells closely followed transformation of the initiated and promoted cells, some problems remained to be solved. The present results extend and complement the previous ones and allow some more definite conclusions to be drawn.

### MATERIALS AND METHODS

### *Cell cultures*

The experiments were carried out with rat embryo fibroblasts prepared from pathogen-free Wistar rats, from our own breeding colony. Primary cultures were prepared from 14-day-old embryos that had been aseptically removed, washed twice in phosphate-buffered saline (PBS) and minced in small pieces. The tissue fragments were dispersed for 5 min in PBS with a magnetic stirring rod and then submitted to the action of 0·25% trypsin, both operations being carried out at 37°C. The supernatants from the first 20-min digestion were pooled after the action of the trypsin had been stopped by the addition of calf serum. The cells were filtered through sterile gauze and centrifuged for 5 min at 1000 rev/min. The cell pellets were resuspended in Dulbecco's growth medium H16 (GIBCO) supplemented with 10% foetal calf serum (GIBCO) and were used to prepare primary cultures. After counting with a Thomas haemocytometer, the cells were diluted to $0{\cdot}5 \times 10^6$/ml and seeded in 10-cm plastic Petri dishes (Falcon Plastics), each dish receiving 10 ml of the cell suspension ($5 \times 10^6$ cells per dish). They were incubated at 37°C in a humidified atmosphere of air with 10% $CO_2$. Every 5 days the cells were collected using a 0·25% trypsin solution and subcultured at $10^5$ cells/ml of medium.

### *Chemicals*

Dimethylbenz(a)anthracene (DMBA) (Fluka Co., Switzerland), benzo(a)pyrene (BaP) (Schuchardt Co., Munich, Germany) and benzo(e)pyrene (BeP) (K and K Laboratories Inc., Plainview, N.Y., U.S.A.) were used as initiators. They were purified before use by thin-layer chromatography on silicagel, using benzene as solvent.

Croton Oil (Cr.Oil) (Schuchardt Co., Munich, Germany) and 12-O-tetradecanoylphorbol-13-acetate (TPA), kindly donated by Professor E. Hecker (Heidelberg, Germany), were used as promoters.

All substances were added to the growth medium in acetone solution. The final concentration of solvent in the medium never exceeded 0·5%, a concentration which did not affect cell growth or morphology.

### *Treatment*

*Initiation.*—One day after seeding, the cells from the third passage (P3) received the initiator: DMBA (Exp. I), BaP or BeP (Exp. II). DMBA was added as a single dose of 0·5 μg/ml of medium for 24 h, BaP and BeP at a concentration of 1 μg/ml of medium for 6 h only. After treatment with initiator, cells were immediately washed with normal medium and reincubated in fresh medium at 37°C until treated with either promoter or solvent. A second series of dishes, containing cells from the same P3, received an initial treatment with solvent only.

*Promotion.*—The promoting treatment started 7 days after the initiation treatment, using either TPA or Cr.Oil added at a final concentration of 0·01 μg/ml of medium. The promoters were added as solutions in acetone at every passage until the end of the experiments. The control cells received the solvent only.

### *Transformation assay*

The effects of the different treatments on the cells, were estimated, at intervals, as shown in Fig. 5. The tests consisted of cloning in liquid medium, the results being expressed by (a) the cloning efficiency (CE), and (b) the transformation efficiency (TE), and of the s.c. inoculation of the cells into newborn rats expressed by (c) the malignancy.

*Cloning efficiency* (*CE*).—Tissue culture cells, when sparsely seeded, tend to develop into colonies (Puck and Marcus, 1955). After trypsinization, 200 cells from each group were suspended in 2 ml of Dulbecco's medium supplemented with 20% foetal calf serum and were plated in 60-mm plastic Falcon Petri dishes and incubated at 37°C in an atmosphere of air with 10% $CO_2$. Ten days later the colonies were fixed in methanol and stained with Giemsa. The percentage of plated cells developing into colonies is indicative of the *cloning efficiency* (*CE*) of the cells.

*Transformation* (*T*).—The transformation in these experiments was recognized by the abnormal morphological characteristics of the colonies, such as a loss of cell orientation and a piling-up of the cells in a random criss-cross pattern (Berwald and Sachs, 1965). The percentage of atypical colonies represented the *transformation efficiency* (*TE*) of the culture.

*Malignancy.*—The malignancy of the cells was determined by inoculation into animals. The treated and control cells from different passages were inoculated s.c. into the cranial region of 2-day-old isologous Wistar rats, each animal receiving 2 or $3 \times 10^6$ cells as a suspension in 0·05 ml of culture medium. The animals were subsequently examined twice a week for palpable tumour formation, for a period of at least 6 months. Systematic histological examination was performed on all tumours; these appeared to be fibrosarcomas.

### RESULTS

### *Cloning efficiency*

In Exp. I (Fig. 1) the CE of the solvent control cells remained low; the CE of the cells treated with DMBA or with DMBA + TPA increased at P32–P36, whereas that of the TPA treated cells increased rapidly at P16. In Exp. II (Fig. 2) the solvent controls and the BaP-treated cells retained a low CE. With the other treatments, the CE showed variation depending on the treat-

FIG. 2.—Cloning efficiency of rat embryo cells during 2-stage carcinogenesis *in vitro*. Initiators: BaP and BeP, promoter: Cr.Oil (= CrO).

ment and on the number of passages. It increased significantly at P49–P54 with the cells treated with Cr.Oil, BaP + Cr. Oil and BeP + Cr.Oil.

### *Transformation*

Transformation of the control cells (or " spontaneous transformation ") occurred after P49 in both experiments (Figs. 3 and 4). Transformation of cells treated with BaP and BeP (Fig. 4) occurred at approximately the same time as that of the controls, whereas transformation of the Cr.Oil-treated cells was slightly delayed.

FIG. 1.—Cloning efficiency (CE) of rat embryo cells during 2-stage carcinogenesis *in vitro*. Initiator: DMBA, promoter: TPA.

FIG. 3.—Transformation of rat embryo cells during 2-stage carcinogenesis *in vitro*. Initiator: DMBA, promoter: TPA.

FIG. 4.—Transformation of rat embryo cells during 2-stage carcinogenesis *in vitro*. Initiators: BaP and BeP, promoter: Cr.Oil (= CrO).

On the other hand, transformation was markedly accelerated for cells treated with DMBA + TPA, BaP + Cr.Oil and BeP + Cr.Oil (Figs. 3 and 4). Transformation of the DMBA-treated and TPA-treated cells was also accelerated, and occurred soon after that of the (DMBA + TPA)-treated cells.

### *Malignancy*

Malignancy of the control cells (or " spontaneous malignant transformation ") was demonstrated at P55 (275 days of culture) for Exp. I (Fig. 5), and at P67 (335 days) for Exp. II (Fig. 5). In contrast, the malignancy of the cells treated with DMBA + TPA and BaP + Cr.Oil appeared respectively at P36 (180 days) and P28 (140 days). Other treatments gave results between these 2 extremes. In Exp. I, the malignancy of the cells treated with TPA and DMBA alone was demonstrated respectively at P40 (200 days) and at P46 (230 days). These transformation times were close to those of (DMBA + TPA)-treated cells (P36) and the difference between the DMBA + TPA treatment and the TPA-only treatment cannot be considered as significant.

In Exp. II (Fig. 5) the malignancy of BaP-treated cells was demonstrated at P46 (230 days); the malignancy of Cr.Oil-treated cells at P51 (255 days) and that of BeP + Cr.Oil and BeP-treated cells at P61 (305 days) of the cultures. These data

FIG. 5.—Transformation of rat embryo cells in tissue culture by chemicals. Experimental protocol showing initiation with DMBA, BaP or BeP followed by promotion with TPA or Cr.Oil. Cell transformation data obtained by cloning in liquid medium: and malignancy from the results of inoculation of cells into newborn animals. * M = number of tumours per total number of animals inoculated. T = transformed clones/100.

TABLE.—*Effect of Initiation and Promotion on the Transformation and Malignancy of Rat Cells in Culture, and on the Acceleration of these Phenomena*

| | Treatment | | | | | | |
|---|---|---|---|---|---|---|---|
| | Initiation | Promotion | $T_{20}$ | $AT_{20}$ | M | AM | $M - T_{20}$ |
| Exp. I | — | — | 255 | — | 275 | — | 20 |
| | DMBA | TPA | 160 | 95 | 180 | 95 | 20 |
| | DMBA | — | 175 | 80 | 230 | 45 | 55 |
| | — | TPA | 170 | 85 | 200 | 75 | 30 |
| Exp. II | — | — | 255 | — | 335 | — | 80 |
| | BaP | Cr.Oil | 120 | 135 | 140 | 195 | 20 |
| | BaP | — | 270 | (−15) | 230 | 105 | (−40) |
| | BeP | Cr.Oil | 145 | 110 | 305 | 30 | 160 |
| | BeP | — | 245 | 10 | 305 | 30 | 60 |
| | — | Cr.Oil | 315 | (−60) | 255 | 80 | (−60) |

$T_{20}$—Culture time (in days) for 20% transformation.
$AT_{20}$—Acceleration of $T_{20} = T_{20\ controls} - T_{20\ treated}$
M—Culture time (in days) for malignancy.
AM—Acceleration of $M = M_{controls} - M_{treated}$.

are of course approximate, since there were variations in the number of animals that developed tumours, stemming presumably from variations in the malignancy of the injected cells. We observed that after the first positive inoculation there was a small number of tumours, but these only became apparent after a long latent period. With subsequent inoculations all animals developed tumours, and the latent period became shorter, even if the number of cells inoculated was lower (Table). The malignant potential of treated cells can thus be estimated from the length of the latent period that precedes tumour development.

### DISCUSSION

### *Cloning efficiency*

Cloning efficiency, as well as the occurrence of morphologically abnormal colonies, are both generally accepted criteria of cellular transformation after treatment with chemical carcinogens (Puck and Marcus, 1955). Our results with rat fibroblasts, however, show no correlation between the appearance of an increase in CE and morphological transformation. For example, CE increased before transformation (with TPA), accompanied transformation or immediately followed it (with DMBA + TPA, BaP, BeP, Cr.Oil, and controls), or increased later (BaP + Cr.Oil, BeP + Cr.Oil) (Figs 1 and 2). In addition, there did not appear to be a correlation between the appearance of an increase in CE and *in vivo* malignancy: CE increased before malignancy (with BeP + Cr.Oil, DMBA, TPA, Cr.Oil), at the same time or immediately after cell malignancy (controls of Exp. I, DMBA + TPA), or much later (BaP + Cr.Oil). CE also either decreased (after treatment with BaP) or remained at the same level throughout the experiments (after BeP and the controls of Exp. II).

These results show that the level of CE does not have the same significance in this type of study as transformation or malignancy, and that different treatments, given either alone or in combination, can influence the CE of rat embryonic cells in different ways which do not appear to be related to the other 2 parameters examined.

### *Transformation and malignancy*

Abnormal colony morphology is considered as a criterion of transformation, especially with studies using Syrian hamster cells (Berwald and Sachs, 1963) in which transformation can vary from 0 to 25% depending on the treatment and on the experimental conditions (Berwald and Sachs, 1965; DiPaolo *et al.*, 1969; DiPaolo, Donovan and Nelson, 1971*a*;

1971*b*; Huberman *et al.*, 1972). Transformation of rat cells has been studied mostly by looking for the appearance of transformed foci (Freeman, Igel and Price, 1975). Current studies show that, after cloning, the frequency with which transformed colonies of rat cells is seen is variable, and that the transformed state is not stable when this frequency is below 15%, although this becomes more stable as soon as this value approaches 20%. Below 15%, normal cells may inhibit the manifestation of transformation (Berwald and Sachs, 1965). The 20% transformation level (T20) then seems to be the critical threshold of transformation for rat cells, above which there is a constant increase. If a line is drawn parallel to the abscissa at the 20% mark on the ordinate (Figs 3 and 4), it will cut the transformation curves at a point representing a number of passages or days in culture. T20 can therefore serve as a reference point for transformation measurements and, despite the possible variations, it would permit a quantitative evaluation of the transforming effects of the treatments. Since control cells also become transformed, one can also calculate the acceleration of transformation (AT20) caused by a compound, by taking the difference between the T20 of the control cells and the T20 of the treated cells ($AT_{20} = T_{20c} - T_{20t}$) expressed in days (Table).

Malignancy reflects the capacity of transformed cells to form tumours after their inoculation into isologous animals. It is therefore possible to calculate, in days, the acceleration of the acquisition of malignancy (AM) as the difference between the time at which the control cells first exhibit malignancy ($M_c$) and the time at which malignancy can first be demonstrated for treated cells ($M_t$); ($AM = M_c - M_t$) (Table).

This manner of presenting the results permits us to examine correlations between transformation (T) and malignancy (M) and between the carcinogenic action of the substances *in vivo* and their action *in vitro*. During these studies, we noted that in 8 cases, cells showed morphological transformation before they exhibited malignancy, but that in 2 cases (BaP-treated and Cr.Oil-treated) morphological transformation was not seen until after malignancy. Secondly, the BeP + Cr.Oil treatment significantly accelerated cell transformation (110 days) but altered the onset of malignancy to a lesser extent (30 days), whereas Cr.Oil-alone retarded transformation (—60 days) and accelerated malignancy (80 days). These data suggest that even though transformation generally precedes malignancy, the 2 phenomena are not necessarily linked as has been shown with guinea-pig cells (Evans and DiPaolo, 1975).

When the results (Table) were analysed as a function of the carcinogenic action of the substances used, we noted that the correlation was good for DMBA and BaP alone or combined with TPA or Cr.Oil. The action of TPA alone *in vitro* is as *in vivo*. Besides its co-carcinogenic activity, TPA exhibits a weak but definite carcinogenic activity (Chouroulinkov and Lazar, 1974; Hecker, 1966, 1968).

The acceleration of transformation by BeP + Cr.Oil suggests that BeP is an initiator, which is in accord with the results already observed *in vivo* (Scribner, 1973), but these results need to be confirmed in further studies. Cr.Oil treatment gave some disturbing results. The acceleration of malignancy by Cr.Oil can be attributed to its weak tumorigenic activity (Boutwell, Bosch and Rusch, 1957). The accelerating action on cell malignancy and its retarding effect on transformation are both suppressed when the cells are pretreated with BeP. This observation, and the fact that TPA has no effect on the CE of the cells initiated with DMBA (Table) indicate that the cells pretreated with DMBA or BeP do not respond to Cr.Oil and TPA like normal cells.

If the action of BaP and BeP as initiators is examined, it seems that the 2 hydrocarbons acted on transformation " sites ", since in both cases Cr.Oil accelerated transformation (T). In contrast, the " sites " of the initiation of malignancy were affected by BaP but not by BeP, since Cr.Oil accelerated malignancy in the first case and not in the second. We may conclude that the sites for transformation are different from those involved in malignancy. Other workers have suggested that tumour promoters act *in vitro* by releasing cells from contact inhibition (Sivak and van Duuren, 1967, 1970).

*Co-carcinogenesis*

Berenblum (1974) recently defined the " basic requirements " for the existence of a 2-stage carcinogenesis process, which we have tried to apply to our tissue culture experiments.

1. The 2 separate actions, initiation and promotion, should not overlap in time.

Our experiments were designed in such a way that the initiation step, lasting 6 or 24 h, was separated from the first promotion step by 7 days (see Fig. 5).

2. Neither action alone should be carcinogenic.

As shown, the results do not appear to satisfy this condition, because of the spontaneous transformation of the cells and the positive reactions obtained with both the " initiators " and the " promoters ". However, one should not forget that the cellular model differs from the animal model because of the earlier " spontaneous transformation " of the control cells. The death of the animal normally prevents one from observing " spontaneous " carcinogenesis in skin cells, and even if this does occur, we do not know the latent period. However, if we stop our *in vitro* experiments earlier, before spontaneous transformation, the cellular model becomes much more analogous to the animal model.

The positive effect of the initiators and promoters is to a certain extent a problem of dosage. Although the initiating doses of DMBA and BaP and the promoting doses of TPA and Cr.Oil are known for mouse skin, we did not know the appropriate doses to use with cells in culture when these experiments were started. The results of Exp. I show that the initiating dose of DMBA was too high; those of Exp. II seem more clear-cut, even if the initiating dose of BaP was still too high.

3. Extending the interval between the 2 actions should not alter the tumour yield.

For tissue culture experiments, this condition should be modified to read: the acceleration of malignancy should not be modified by the interval between the 2 treatments. In an earlier experiment with BaP and TPA (Lasne *et al.*, 1974), promotion was begun 35 days after initiation, and the results were similar to those presented here.

4. The ultimate tumour yield should be quantitatively related to the dose of initiator, while the efficacy and the speed of tumour induction should be determined by the promoter.

To respond to this condition, one must see to what extent a qualitative relationship exists between the cutaneous lesions arising during carcinogenesis *in vivo* and the cellular modifications seen in culture.

On mouse skin, benign tumours develop which may later evolve into malignant tumours, especially if the treatment is continued. However, malignant tumours can also develop directly. Similarly, in cell culture, morphological transformation usually appears before the cells can be shown to be malignant, but the latter can sometimes be demonstrated prior to morphological transformation (Table).

According to the 4th condition listed as a requirement for 2-stage carcinogenesis, the number of tumours would be determined by the initiation and the latent period for tumour formation by promotion. In culture, initiation actually influences both the amount of cell transformation

(expressed as a percentage) and the number of malignant cells, which plays an important role in tumour development. Promotion *in vitro* affects both the latent period, and the acceleration time.

The results presented here show that the malignant transformation of cells in 2 stages is reproducible in tissue culture, as has been demonstrated in another experimental system using mouse cells (Mondal, Brankow and Heidelberger, 1976). These authors concluded that the promoters were not acting by selecting transformed cells and that it should be possible to study the mechanisms involved in initiation and promotion in an experimental *in vitro* system more simply than in the whole animal: cultured cells can be considered as a model system that will facilitate further investigations on the carcinogenic or co-carcinogenic actions of environmental agents.

We wish to acknowledge the excellent technical assistance of Mrs F. Chauveheid. We are indebted to Dr P. L. Grover (Chester Beatty Research Institute, London) for advice.

### REFERENCES

Berenblum, I. (1941) The Mechanism of Carcinogenesis: a Study of the Significance of Cocarcinogenic Action and Related Phenomena. *Cancer, Res.*, **1**, 807.

Berenblum, I. (1974) *Carcinogenesis as a Biological Problem*. Oxford: North Holland.

Berenblum, I. & Shubik, P. (1947) A New Quantitative Approach to the Study of the Stages of Chemical Carcinogenesis in the Mouse's Skin. *Br. J. Cancer*, **1**, 383.

Berenblum, I. & Shubik, P. (1949) An Experimental Study of the Initiating Stage of Carcinogenesis and a Re-examination of the Somatic Cell Mutation Theory of Cancer. *Br. J. Cancer*, **3**, 109.

Berwald, Y. & Sachs, L. (1963) *In vitro* Cell Transformation with Chemical Carcinogens. *Nature, Lond.*, **200**, 1182.

Berwald, Y. & Sachs, L. (1965) *In vitro* Transformation of Normal Cells to Tumor Cells by Carcinogenic Hydrocarbons. *J. natn. Cancer Inst.*, **35**, 641.

Boutwell, R. K., Bosch, D., Rusch, H. P. (1957) On the Role of Croton Oil in Tumor Formation. *Cancer Res.*, **17**, 71.

Chen, T. T. & Heidelberger, C. (1969) Quantitative Studies on the Malignant Transformation of Mouse Prostate Cells by Carcinogenic Hydrocarbons *In vitro*. *Int. J. Cancer*, **4**, 166.

Chouroulinkov, I. & Lazar, P. (1974) Action Cancérogène et Cocancérogène du 12-O-tétradécanoyl-phorbol-13-acétate (TPA) sur la Peau de Souris. *C. R. Acad. Sci. Paris*, **278-D**, 3027.

DiPaolo, J. A., Donovan, P. J. & Nelson, R. L. (1969) Quantitative Studies of *In vitro* Transformation by Chemical Carcinogens. *J. natn. Cancer Inst.*, **42**, 867.

DiPaolo, J. A., Donovan, P. J. & Nelson, R. L. (1971*a*) Irradiation Enhancement of Transformation by Benzo(a)pyrene in Hamster Embryo Cells. *Proc. natn. Acad. Sci. U.S.A.*, **68**, 1734.

DiPaolo, J. A., Donovan, P. J. & Nelson, R. L. 1971*b*) Transformation of Hamster Cells *In vitro* by Polycyclic Hydrocarbons without Cytotoxicity. *Proc. natn. Acad. Sci. U.S.A.*, **68**, 2958.

Evans, C. H. & Dipaolo, J. A. (1975) Neoplastic Transformation of Guinea-pig Cells in Culture Induced by Chemical Carcinogens. *Cancer. Res.*, **35**, 1035.

Freeman, A. E., Igel, H. J. & Price, P. J. (1975) Carcinogenesis *In vitro*. I. *In vitro* Transformation of Rat Embryo Cells: Correlations with the Known Tumorigenic Activities of Chemicals in Rodents. *In Vitro*, **11**, 107.

Friedwald, W. F. & Rous, P. (1944) The Initiating and Promoting Elements in Tumour Production. *J. exp. Med.*, **80**, 101.

Hecker, E. (1966) Die Cocarcinogene Wirkung der Phorbolester. 17th *Colloquium, Ges. Physiol. Chem.*, 105.

Hecker, E., (1968) Cocarcinogenic Principles from the Seed Oil of *Croton tiglium* and from other Euphorbiaceae. *Cancer Res.*, **28**, 2338.

Huberman, E., Kuroki, T., Marquardt, H., Selkirk, J. K., Heidelberger, C., Grover, P. L. & Sims, P. (1972) Transformation of Hamster Embryo Cells by Epoxide and other Derivatives of Polycyclic Hydrocarbons. *Cancer Res.*, **32**, 1391.

Lasne, C. (1973) Thesis (Université, Paris VI).

Lasne, C., Gentil, A. & Chouroulinkov, I. (1974) Two-Stage Malignant Transformation of Rat Fibroblasts in Tissue Culture. *Nature, Lond.*, **247**, 490.

MacPherson, I. (1970) The Characteristics of Animal Cells Transformed *In vitro*. *Adv. Cancer Res.*, **13**, 169.

Mondal, S., Brankow, D. W. & Heidelberger, C. (1976) Two-Stage Oncogenesis in Cultures of C3H/10Tl/2 Cells. *Cancer Res.*, **36**, 2254.

Puck, T. T. & Marcus, P. I. (1955) A Rapid Method for Viable Cell Titration and Clone Production with HeLa Cells in Tissue Culture. *Proc. natn. Acad. Sci. U.S.A.*, **41**, 432.

Scribner, J. D. (1973) Tumor Initation by Apparently Noncarcinogenic Polycyclic Aromatic Hydrocarbons. *J. natn. Cancer Inst.*, **50**, 1717.

Sivak, A. & van Duuren, B. L. (1967) Phenotypic Expression of Transformation: Induction in Cell Culture by a Phorbol Ester. *Science, N.Y.*, **157**, 1443.

Sivak, A. & van Duuren, B. L. (1970) A Cell Culture System for the Assessment of Tumor-promoting Activity. *J. natn. Cancer Inst.*, **44**, 1091.